# Exploring Causes of Depression and Anxiety Health Disparities (HD) by Examining Differences between 1:1 Matched Individuals

**DOI:** 10.3390/brainsci8120207

**Published:** 2018-11-28

**Authors:** Emil N. Coman, Helen Z. Wu, Shervin Assari

**Affiliations:** 1 UConn Health Disparities Institute, University of Connecticut Health Center, Hartford, CT 06106, USA; 2 Department of Psychiatry, University of Connecticut Health Center, Farmington, CT 06030, USA; zwu@uchc.edu; 3 Department of Psychiatry, School of Medicine, University of Michigan, Ann Arbor, MI 48109, USA; assari@umich.edu

**Keywords:** matching, latent difference score, health disparities, causality

## Abstract

Poor comparability of social groups is one of the major methodological problems that threatens the validity of health disparities (HD) research findings. We illustrate a methodological solution that can additionally unpack the mechanisms behind differential effects on depression and anxiety. We describe racial/ethnic differences in the prevalence of depression and anxiety scores between Black and White women using classic methods, and then we illustrate a 1:1 matching procedure that allows for building of individual-level difference scores, i.e., actual HD difference score variables, for each pair of comparable participants. We compare the prevalence of depression disorder between Black and White young women after matching them 1:1 on common socio-economic characteristics (age, employment, education, and marital status). In essence, we follow matching or stratification methods, but make a step further and match cases 1:1 on propensity scores, i.e., we create Black–White ‘dyads’. Instead of concluding from plain comparisons that 11% more White young women (18–30 years old) report a depressive disorder than Black young women, the matched data confirms the trend, but provides more nuances. In 27% of the pairs of comparable pairs the White woman was depressed (and the comparable Black woman was not), while in 15% of the pairs the Black woman was depressed (and the comparable White woman was not). We find that Black-to-White disparities in neighborhood disorder do not predict depression differences (HDs), while such an effect is evident for anxiety HDs. The 1:1 matching approach allows us to examine more complex HD effects, like differential mediational or resilience mechanisms that appear to be protective of Black women’s mental health.

## 1. Introduction

Understanding the underlying causes of health disparities (HD) is a major research objective in the US and abroad, because it promises to uncover efficient solutions for health equity [1,2]. Finding the evidence for actual *causes* of HDs can provide researchers, healthcare providers, and policy makers with insights for policy and actions [3] that can reduce and even eliminate health inequities.

HDs are differences in health outcomes between groups that are avoidable; this implies that if one knows what causes them, one can avoid, or at least reduce them. When such disparities in health are not reduced even when causal mechanisms are understood, HDs qualify as inequities, and hence they are unfair [4]. To decide fairness and equity however, policy makers try to infer what would happen to members of a disadvantaged racial/ethnic group, had they been members of the privileged group; such imaginary exercises however have to assume that members of different R/E groups are ‘exchangeable’, or that one can infer what could have happened to a person from one group had they switched roles with an ‘identical’ person from another group. We provide a method that puts in practice this imaginary ‘what if’ (or counter-factual (CF) [5]) exercise that increases comparability and potentially reduces confounding [6], by directly matching 1:1 participants on all background factors, then re-assessing the range of differences in health outcomes.

Health disparities have been documented in mental health outcomes in general in the US, and in depression in particular. Research shows that fewer Blacks in the US report depression than Whites [7] or Hispanics/Latino/a [8]. Blacks in the US on the other hand may show higher rates of anxiety than Whites, although such differences are thought to be partially confounded by sociodemographic indicators [9]. It is possible that Blacks may differentially experience stronger depressive symptoms as responses to stimuli like physical symptoms of illness [10], but may cope better with other stressful events, because they are accustomed to adversity because of belonging to a minority [11,12]. Even if less prevalent in Blacks, metal health symptoms may have stronger health consequences in Blacks than other racial/ethnic (R/E) groups [13].

The environment clearly affects health [14,15], and it may contribute differentially to health outcomes, hence increasing inequalities [16]. Neighborhood conditions represent opportunity structures [17], and hence can become a form of environmental stress or social adversity [18], which has also been conceptualized as a form of toxic stress [19] or life adversity [11,12]. Neighborhood conditions have long-lasting social consequences [20], primarily by determining intermediate outcomes, like educational attainment [21,22]. These effects can accumulate over the life course [23] and can lead to disparate health effects like ‘accelerated aging’ [24]. We explore a method that can gauge how disparities in neighborhood conditions may potentially affect disparities in mental health, depression primarily, and anxiety secondarily.

Modern HD research takes on the task of examining causes of existing HDs by comparing population estimates of health outcomes of interest between racial/ethnic (R/E) groups, or other populations, first as they are, and then while controlling for relevant covariates. While such group differences represent a first approximation of disparities, HD researchers ultimately aim to identify their causes and hence recommend corrective courses of action. One method promising to provide causal conclusion is the well-known potential outcomes (PO) approach to understand HDs [25], which compares alternative POs for the same person, under different conditions.

In HD research we ask whether the same person would experience a better/worse outcome, had s/he changed her/his race/ethnicity, while everything else is kept equal, i.e., *ceteris paribus*. For example, in a recent court case the parties made such counterfactual (CF) assertions, that Asian Americans would have a better chance of being admitted to an elite university, had they been White, or Black, or Hispanic [26]. Such mental exercises can be justifiably envisioned for CF ‘gender switching’, because gender is assigned randomly at birth [27]. Race/ethnicity however is not assigned randomly, so a mere difference between average scores cannot be seen as a measure of a (causal) health disparities effect. If people of different races/ethnicities (R/E) were truly ‘exchangeable’ (i.e., similar, see [28] p. 159), then the mere difference between group means in an outcome Y, ${\bar{Y}}_{B}$ − ${\bar{Y}}_{W}$, could be deemed a ‘causal effect’ of being Black (vs. White, e.g.,), but such exchangeability is not realistic. Researchers hence need additional tools to gauge ‘true’ (i.e., causal) HD effects, because the ‘everything else being equal’ assumption requires some form of ‘controlling’ for, or stratifying on several factors. One common way to control for pre-existing differences is to compare the focal outcome between subgroups that have the same levels of covariates (are balanced on them), [29], by matching for instance individuals from both groups on education and employment, and then comparing several sub-groups that were made similar by statistical means (see [30] or [31] for illustrations using propensity matching).

The statistical controlling option known nowadays as ‘propensity matching’ was suggested long ago by Cochran [32], exactly as a 1:1 match initially, done in ‘the hope of securing a more accurate comparison’ (p. 256). We propose that such a 1:1 matching can additionally unravel causal mechanisms responsible for health disparities. If one matches 1:1 say Black and White participants, on relevant demographics and known socio-economic indicators, then an individual HD (difference) score on an outcome Y can be created for each matched pair *i*, like HD_i_ = Y_iB_ − Y_iW_, and then this new variable can be analyzed in terms of co-variability with other such difference scores. The estimate of the health disparity in an outcome Y will then become the *average of* such varying *differences*, $\bar{\mathrm{HD}}$_i_, instead of the more limited *differences between averages* ${\bar{Y}}_{B}$ − ${\bar{Y}}_{W}$, as it is commonly done. We show how to create such ‘exchangeable’ pairs by matching 1:1 as close as possible, on a number of background factors, after reviewing relevant health disparities literature.

## 2. Conceptual Models for Health Disparities

Social disadvantage or low social class are known as ‘social sources’ of HDs [33]. In fact HDs are seen as differences in health resulting from social forces, not due to unavoidable biological differences [34]. Social determinants of health like neighborhood poverty, relative income inequality, poor housing, and segregation can become ‘place-based drivers’ of disparities in health [34]. In essence societies ‘act’ by sorting individuals in resource-rich and resource-poor neighborhoods, and then individuals themselves make choices (to the extent they have such choices) to stay in, or move between, different places [35], which can shape their life-course trajectories and health.

**Figure 1:**
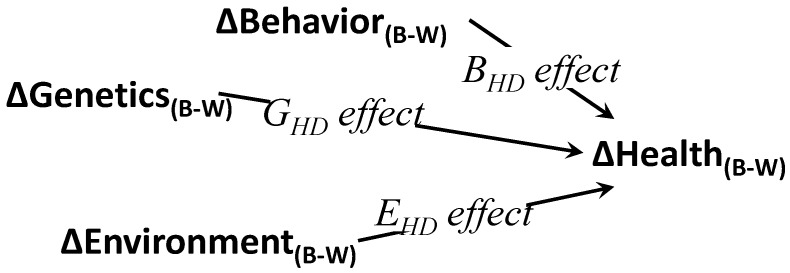
Simplified conceptual model of major causes of Health Disparities (HD). Notes: G_HD_, E_HD_, and B_HD_ are the effects of the differences in genetics, environment, and individual behavior on differences in health (or health disparities, HD); B: Black, W: White groups.

We propose that what is missing from analytical modeling of HDs is the direct specification of HDs as a distinct *outcome* (or ‘dependent variable’) in models, either logic, conceptual or theoretical, which can then be turned into a variable at the analysis stage. Many HD writings point to presumed causal mechanisms behind HDs. Health inequities are seen to *arise from* “social, economic, environmental, and structural disparities that contribute to intergroup differences in health outcomes” ([35], p. 99). A HD simplified causal model is shown in Figure 1, which crystalizes an array of HD theoretical models, and promises to reveal causal forces leading to HDs. The relative contribution of these factors is important; research for example has pointed to one’s zip code as being more important to one’s health than one’s genetic code [36].

## 3. Methods

### 3.1. Study Setting and Samples

The data come from a larger study on stress and substance use in young women, which was conducted between November 2006 and January 2012 in Southeast Texas [37,38], and collected longitudinal data on stress its correlates; a limited dataset are posted online at the Harvard Dataverse [39] (https://dataverse.harvard.edu). Participants were selected from patients attending one of six University of Texas Medical Branch (UTMB) community-based family planning clinics; we use the baseline data for analyses: *n*_White_ = 92, *n*_Black_ = 145. These clinics serve primarily low-income women with average annual income below $6000. Inclusion criteria were: being (1) female, (2) not currently pregnant, (3) aged 18 and over, (4) non-Hispanic white, non-Hispanic black, or Hispanic, (5) able to speak English or Spanish, and (6) able to consent; all participants provided informed consent. This study was approved by the UTMB’s Institutional Review Board.

### 3.2. Measures

Depression was measured as using the Composite International Diagnostic Interview (CIDI)—World Health Organization version [40]. Assessment of mental health disorders followed the definitions and criteria of the Diagnostic and Statistical Manual of Mental Disorders, 4th Edition (DSM-IV) [41]. Anxiety was measured using five items from Carver’s BIS scale [42] to explain a behavioral avoidance (or inhibition) system (BIS) which is said to regulate aversive motives, in which the goal is to move away from something unpleasant. Responses to BIS items are obtained on a 4 point scale ranging from 1 ‘very true for me’ to 4 ‘very false to me’. Five BIS items loaded on a distinct BIS factor according to a principle factor analysis with oblique rotation; their internal reliability Cronbach’s alpha was 0.696.

Neighborhood disorder was measured using ten items [43] assessing how residents perceived problems related to safety and signs of physical neglect in their neighborhood (e.g., poor sidewalks and broken curbs, vandalism). Responses to each question were *z* scored and averaged across questions to create individual-level summary measures of perceived neighborhood social cohesion and disorder (Cronbach’s alpha was 0.83) [38,43].

Covariates used were: age (continuous, range 18–30), marital status (married, co-habitating, not married with boyfriend, not married without boyfriend), education (less than-, with-, or more than high school), and employment (unemployed, homemaker, part-time, fulltime).

### 3.3. Analytical Methods

We follow the logic behind the popular ‘matching’ methods, known also as principal stratification, which combines matching factors into a global score, built as a continuous probability (or ‘propensity’) to belong to one group of interest, in our case Black women, versus the reference group, here White women. This is simply done by regressing the binary grouping (Black vs. White, or ‘treated’ vs. controls [44] more generally) on the variables that can predict belonging to the group of interest (vs. reference), and saving the predicted probabilities from the logistic model (note that, if race was randomized, no variable could predict belonging to one or another race). Practically then, one generates a probability score, of belonging to the group of interest, Black women (vs. the comparison group, i.e., White women), using all relevant factors as predictors in a simple logistic regression. It is known however that such matching is never perfect, minorities usually having fewer cases with higher education, or better employment, for example, than White participants.

Since its inception [32,45], the ‘propensity’ matching method has expanded tremendously, less so however in its 1:1 original matching form, and more so in the matching into comparable strata (or clusters). We propose here a natural HD extension of this methods, which results in a ‘matched pairs’ data structure ([46], chapter 9) that allows for additional testing options.

The common matching approach can be seen as a way of ordering the data on all matching factors, and then pairing up strata in a group (Blacks, or the ‘treated’) to comparable strata in the reference group (Whites, or the ‘controls’). In HD research, when health differences between certain populations are hypothesized, one can simply sort (or stratify) the data ascendingly (or alphabetically, for text categorical variables) by all relevant factors, for example by age and education, and then within each sub-group *g* with similar ages and education, compare the two populations (White and Black women, in our case), which will yield *g* such health disparity quantities HD_g_ = Y_gB_ − Y_gW_. These group estimates can then be averaged out to yield a global HD score. If one uses for illustration three age and three education categories, total *g* is 3 × 3 = 9, as shown in Table 1. Within 5 of these same-age-and-education sub-groups, more White women were depressed, whereas in 4 such subgroups, more Black women were depressed, which are nuances that are lost when one simply reports an overall average White-Black difference, as the (weighted) average of 11% difference would tell.

We basically propose to extend this logic to compare 1:1 individual White and Black women in similar ‘groups’, technically dyads, made up of one participant from each group, which will yield a more detailed HD picture. Guided by the conceptual model in Figure 1, our approach is to directly compare truly comparable individuals, after matching them 1:1 on background factors, like socio-economic status. We do so by aligning disadvantaged (minority) and reference (majority) participants with nearly identical ‘propensity’ scores. This simple step allows one to literally build HD scores for each ‘dyad’ of comparable cases, and then describe and analyze the variability of these ‘dyadic’ HD scores, hence directly evaluating the *causes* of health disparities in depression (or anxiety).

First, we select the matching variables, the potential confounders that can be associated with both the racial/ethnic grouping and the study outcomes (depression and anxiety). These were 4 demographic and social factors: age, education, employment and marital status (income had missing values for more than half of the sample, so was not useful). Second, we created propensity scores, i.e., probabilities of ‘being a Black woman’ (vs. White) from a logistic regression of the Black/White binary ‘outcome’ on four predictors: age, education, employment, and marital status (*logit* Stata [49] command, with three predictors being categorical). Third, the saved probability values were used to match 1:1 white and Black women (data is shown in the online appendix bit.ly/1to1depression): 61 pairs were matched 1:1. One can then compare Black and White women on any outcome, within either the unmatched (145_B_ vs. 92_W_) or the matched sub-samples (61_B_ vs 61_W_).

Because of the 1:1 matching into comparable ‘dyads’, akin to twins (or parent-child, or spousal dyadic designs), the data can now be re-shaped or re-organized such that any outcome (like depression and anxiety) or predictor (like neighborhood disorder) become ‘repeated’ variables, one each for the White and for their matched Black counterparts, hence the number of ‘cases’ now is as many as the matched dyads, *n* = 61. This ‘double’ data resembles in many ways a ‘pre’ and ‘post’ repeated measures design, where ‘pre’ is simply the reference group, here chosen to be the White women. This simple implementation led us to investigate actual ‘change’ scores, which in our dyadic setup represent ‘difference scores’. Obviously, such difference scores can be computed by hand in the raw data after matching, for any Black/White pair, so one can then investigate the reasons for such varying differences, across the 61 dyads, or to what extent larger such differences are predicted by (for example) differences in neighborhood conditions, like neighborhood disorder. This moreover opens up the option of building health disparities scores as latent difference scores (LDS [50,51]). One can therefore directly investigate the sources of the variability in the HD scores, and more importantly explore causes of HDs in models of increasing complexity.

Notably also, to test for the statistical significance of the differences between the White and Black matched cases, one can now use ‘paired’ tests, like the paired McNemar chi-squared test for binary outcomes, instead of independent samples test, like the plain chi-squared test, as one would, with the original ‘mixed’ (un-matched) cases.

## 4. Results

We report the proportions, means, standard errors, and *p* values for Black/White differences for the key outcomes and descriptives, in Table 2, for the initial sample of women. The initial racial/ethnic (R/E) groups differed significantly in marital status, with more white women in the unmarried and cohabitating categories, and more Black women in the not married or living with a boyfriend categories. Black women live in neighborhoods with more problems, yet they seem to experience less anxiety and depression, which has been called the Black–White paradox [52,53].

The matching process worked well, indicated for instance by the fact that marital status, which, as shown in Table 2, differed initially significantly between Black and White women, became now similarly distributed in the two groups of the matched women (χ^2^(3) = 0.406, *p* = 0.939; the un-matched sub-groups remained different on marital status).

The overall results in terms of depression HDs are summarized in Table 3, where the estimated percentages of women reporting depression and the White–Black differences are shown: the 11% initial estimate, and the 12% revealed after 1:1 matching.

The 1:1 matching turns the testing of HDs in depression (as a binary outcome) from an independent samples test one into a paired samples test, i.e., from a chi-squared test of independence between White and Black women into a McNemar’s chi-squared test, which in essence weighs the number of two types of ‘opposite’ pairs (the diagonal cells in Table 4). The McNemar test is comparing the number of pairs in which a Black woman was depressed and the comparable (matched) White woman was not (a difference score of 1_B_ − 0_W_ = 1, with 0 being non-depressed, and 1 depressed) to the number of pairs with the opposite pattern, i.e., scores for which 0_B_ − 1_W_ = −1. The McNemar’s chi-squared test ignores equality scores, i.e., those for which no difference is evident (whether both are 0_B_ & 0_W_ or both are 1_B_ & 1_W_, see Table 4). We had 9 cases with 1_B_-0_W_, 18 with 0_B_-1_W_ and 34 with Black-White equalities in terms of depression (either both 0’s, or both 1’s), which the McNemar test sees as not a decisive directional difference between the patterns of paired scores (*p* = 0.161).

One can read Table 4 as saying that of the 40 non-depressed White women, most (31, or 78%) have a Black counterpart with the same outcome, and some are instead depressed (9, or 23%). Of the 19 depressed White women however, most (16, or 84%) have a Black ‘pair’ who is not depressed, and only few (3, or 25%) have the same outcome as them. Overall, 58% of all pairs are ‘concordant’ (53% + 5%), and the 27% plus 15% are discordant, with different depression outcomes: the relative size of these two discordant subgroups decides whether we have significant disparities or not.

The pairing of data allows one to literally read the ‘what if’ as a ‘change’ in outcome with a ‘change’ in one’s race/ethnicity: if a White woman could become (statistically at least, or in a possible alternative world) Black, would she still be (non-)depressed, or would she change her status: this ‘what if (‘*ceteris paribus*’ however) question is the key causal question in health disparities that most other methods cannot directly address. In our case, of the non-depressed women who ‘started as White’ (at the ‘pre’ or ‘baseline’, in the ‘change’ reading of paired data commonly analyzed by McNemar tests), most (78%) would still be non-depressed if they ‘became Black’, while fewer would become depressed (23%). On the other hand, if the depressed White women could ‘become Black’, most would become non-depressed (84%), and only a few would stay depressed (16%). Note that unlike the pairing that is achieved by time matching, when one records repeated scores for the same person, the pairing based on socio-demographics is reciprocal, i.e., one can also read what would happen to a non-depressed (or depressed) Black woman, had she ‘turned White’.

Similarly, to test differences or disparities between Black and White women in continuous outcomes, one can now simply use paired *t*-tests (or better latent change score (LCS) models [50]) instead of independent samples *t*-tests, which for neighborhood disorder yields a *t*(20) = −1.985, *p* = 0.061, and for anxiety *t*(23) = 0.574, *p* = 0.574.

The key finding therefore is that whereas initially depression appeared to be (nearly) significantly more prevalent in White than Black women, this 10.8% points difference was likely a slight underestimate by about 1% points (according to the 1:1 matching method), and by 4.2% points (according to the classic propensity score matching by strata). This difference is due to including in the ‘blind’ or raw comparison White and Black women who are not truly comparable (because one could not randomize race). When the ‘un-matched’ cases are taken out, the initial advantage of Black women increases. This is further confirmed by the fact that the ‘un-matchable’ Black women (*n* = 83) had a similar depression rate (18.1%) as the un-matched White sub-group of women (*n* = 31, 22.6%).

We present in Table 5 comparable results for the classic (‘blind’) group comparison, the 1:1 matching, a logistic regression with the four demographic background factors as covariates, as well as a propensity score matching analysis (using Stata’s *psmatch2* [54]), and a clustered logit regression (an alternative to 1:1 matching, by building off clusters of more than 1 woman from each group; 15 total clusters were built, matched closely on the propensity scores). The results of the matching methods are comparable, yet the 1:1 method allows for further analytic insight, like regressing some HD scores on other HD scores, which we briefly report.

When we regressed the HD depression White-Black difference scores on the neighborhood HDs, the effect was not significant, (β = 0.196 *p* = 0.314, see online appendix for details bit.ly/1to1depression; we provide both Mplus [55] and AMOS [56] input and output for the LDS models; the binary nature of depression was ignored in this exploratory illustration). The regression of the HD anxiety latent difference score (LDS) on the neighborhood HDs yields a significant (standardized) effect: β = 0.642 (*p* < 0.001, *R*^2^ = 0.412), hence a sizable variability portion in anxiety HDs (41%) is explained by the Black vs. White differences in neighborhood disorder: larger HDs between White and Black women in neighborhood conditions lead to into larger HDs in anxiety.

## 5. Discussion

We presented an intuitive way of matching 1:1 individual participants from two racial/ethnic (R/E) groups which allows for testing directly what explains the varying size of health disparities scores (HDs) in health outcomes. This method turns the common HD estimate ${\bar{Y}}_{B}$ − ${\bar{Y}}_{W}$ (*difference between averages*) into an average $\bar{\mathrm{HD}}$_iY_ = Y_iB_ − Y_iW_ (*average of differences*) from matched variables Y_iB_ and Y_iW_. The new HD_iY_ variable allows one to ask what *causes* such varying difference/disparities scores, by regressing it on other differences, like socio-economic condition HDs. Directly exploring causes of HDs in comparable minorities (vs. majority or reference groups) can be easily done by first matching 1:1 individuals from both groups on factors like age, income, employment and education.

We found a HD of about 10.8% points, such that fewer Black women seemed to experience depression than White women. If one compares *truly comparable* (‘exchangeable’) Black to White participants, this difference is similar, of about 12% points, but the 1:1 matching moreover allows for asking questions about what actually *causes* health disparities in depression or other mental health outcomes.

Our approach reveals that even when HDs exist overall between two groups, there is a range of such individual HDs, such that even if ‘the typical individual’ from a disadvantaged racial/ethnic group experiences worse outcomes, some members of that group may exhibit a health benefit when compared to reference individuals who are similar to them. Beyond simply aiming to document HDs, our illustration shows that real life is more complex than mere average effects and effect sizes, and that nuanced investigations of individual differences are worth pursuing. Even if research reports that the population average HD in an outcome was not significantly different from zero, patient-level health disparities may in fact exist for individual patients [57]. In other words, case-specific and population-level causal HD statements can be at odds, for some individual cases, which is not unusual [58]: the 1:1 matching makes these cases visible.

### 5.1. Limitations

This simple matching procedure allows one to begin answering new health disparities research questions, much closer aligned to the *ceteris paribus* ‘what if’ question (see Marshall [59], as cited in [60]). It brings to the forefront directly the issue of ‘exchangeability’ from the causal inference literature, simplified in language by Judea Pearl in recent writings. Pearl calls two groups exchangeable if they resemble each other in terms of “all characteristics that have bearing on the response variable” ([47], p. 196). Our approach does the next natural step beyond merely imagining that people or patients from two-groups hypothetical switched places, it allows for individual group members to (statistically) swap positions (in some virtual causal world, or in an ideal ‘social space’ [61]), after being matched on relevant characteristics). The conclusions are limited of course to the observed (measured) matching factors, so full exchangeability that also covers unobserved confounders is not addressed here.

Examining more directly cause–effect processes involved in health disparities however requires a more complex causal inference approach that is still in development [62,63,64]. The decision of what to control for, or match on, is not statistical, but causal (conceptual/theoretical [65]), and hence researchers can always debate why some covariates were included or excluded. Similar to selecting factors for stratification or propensity matching, including in the matching pool intermediate effects on the path from the grouping (race/ethnicity in our case) unto the outcome may introduce additional bias [66], and education, income, and employment have been proven at times to act as mediators; at the opposite end, including as many variables as available in a large pool of ‘independent variables’, as is often done [67], can bias the causal effects sought after in health disparities research, when some of them are mediators, because doing so statistically ‘blocks’ natural causal effects to the outcome [68].

### 5.2. Extensions

More formal ways of gauging the actual size of HDs besides a plain difference between averages ${\bar{Y}}_{B}$ − ${\bar{Y}}_{W}$ become possible when one examines the range of possible HD_i_ individual scores Y_iB_ − Y_iW_ that the 1:1 matching approach we illustrated makes visible. Kessler & Greenberg [69] for example proposed a measure of aggregate pair-wise differences, the sum of squares of difference scores, whose benefits should be further explored. Simple and more complex structural models of the size of discrepancies [70] can be explored, built around the latent difference/change scores made possible by the 1:1 pairing [50,71]; such models can examine differential protective or detrimental effects posited by recent health disparities theories, like the diminished health returns [72]. Further investigations are also needed to unpack the mechanisms [73] behind the ‘Black–White paradox’ in mental health [52], which was confirmed in our data.

## Figures and Tables

**Table 1:** Percent depressed in the White–Black women data within age & education categories.

	White%	*N*_W_	Black%	*N*_B_	∆_W-B_
Age 18–20 years					
<High school	26%	19	0%	13	26%
High school	39%	18	3%	30	36%
>High school	50%	2	0%	2	50%
Age 21–24 years					
<High school	29%	7	29%	17	−1%
High school	29%	14	11%	19	18%
>High school	0%	6	38%	8	−38%
Age 25–30 years					
<High school	0%	3	26%	19	−26%
High school	38%	8	25%	20	13%
>High school	36%	14	40%	15	−4%
**Entire sample**	**30%**	**91**	**19%**	**143**	**11%**

Notes: For this age × education matching example the test of depression prevalence difference yields χ^2^(8) = 15.351, *p* = 0.053; this 3 × 3 example was purposefully built to allow readers to compare to and expand into a formal structural causal modeling (SCM [47]) analysis, as done by Kaufman & Kaufman (see their example in Appendix 2, Table A2.1 [48]).

**Table 2:** Descriptives of the sample and the key measures, for the two racial/ethnic groups.

	White (%)	*N*	Black (%)	*N*	All (%)	*N*	*p*-Values
Total		92		145		237	
Employment^M^							0.165
Unemployed	38.6	34	45.8	66	43.1	100	
Homemaker	13.6	12	7.6	11	9.9	23	
Part-time	20.5	18	13.2	19	16.0	37	
Fulltime	27.3	24	33.3	48	31.0	72	
Education							0.523
0 < Grade < 12	32.6	30	33.8	49	33.3	79	
Grade = 12	43.5	40	48.3	70	46.4	110	
Grade > 12	23.9	22	17.9	26	20.3	48	
Marital status							<0.001
Married	**20.7**	19	7.6	11	12.7	30	
Co-habitating	**32.6**	30	16.6	24	22.8	54	
Not married w/boyfriend	21.7	20	**51.0**	74	39.7	94	
Not married w/o boyfriend	25.0	23	24.8	36	24.9	59	
Means	White	SEs	Black	SEs	All	SEs	*p*
Age	22.59	0.38	23.11	0.30	22.24	0.35	0.280
Neighborhood disorder	−0.15	0.08	0.18 *	0.07	0.04	0.06	**0.002**
Anxiety	14.60 *	0.29	13.73	0.20	13.91	0.22	**0.014**
Depression (%s)	*29.7%* ^†^	8.8%	18.9%	4.7%	23.1%	4.4%	*0.058*
Depression (odds)	*0.42* ^†^	0.10	0.23	0.05	0.30	0.05	*0.058*

Notes: Percentages and counts for categorical variables, and means and standard errors (SE) for continuous variables; *p* values for *χ*^2^ test (categorical) and independent samples *t*-tests (continuous) Black vs. white comparisons; * (and **bold**): significantly higher value (*p* < 0.050); ^†^ (and *italics*): *p* < 0.100.

**Table 3:** Percent depressed in the original White–Black women data, and in the 1:1 matched data.

Percent Depressed	White %	Black %	∆_W-B_	*p*
*N*_B_ = 143, *N*_W_ = 91	30%	19%	11%	0.056
1:1 *N*_B=W_ = 59	32%	20%	12%	0.161

Notes: ∆_W-B_ is the difference between White and Black women; *p* are the significance values of the comparison tests.

**Table 4:** McNemar test of the paired depression data.

Matched Pairs	Black not Depressed	Black Depressed	Total
White not depressed	78% ^‡^	23%	100%
	31 ^‡^	9	40
	66% ^‡^	75%	
	53% ^‡,T^	15% ^T^	
White depressed	*84%*	16% ^‡^	100%
	16	3 ^‡^	19
	34%	25% ^‡^	32% ^A^
	27 ^T^	5% ^‡,T^	
*Total*		20% ^A^	
	47	12	59
	100%	100%	
			100%

Notes: McNemar’s paired test of differences: *χ^2^* (1) = 1.960, *p* = 0.161 (if one ignores here the dependence/pairing, the expected counts would be 32, 8, 15, 4, and *χ^2^* (1) = 2.111, *p* = 0.550); ^A^ average percentages of depressed women in each White and Black group; ^T^ indicates percentages are of the total sample N; ^‡^ shows pairs similar in depression status.

**Table 5:** Black vs. White women comparisons of percent depressed using classical and modern tests.

Test: Category	Chi Squared	McNemar 1:1 Matched in 61 Dyads	Logistic Regression with Covariates	Logit Regression (For Un-Matched)	Propensity Matching	Clustered Logit (Matched in 14 Clusters)
White women (*n*_DW_/*N*_W_)	27/91	19/59	26/88	7/31	26/87	26/88
White women (%)	29.7%	32.2%	46.9% ^A^	22.6%	29.9%	29.5%
Black women (*n*_DB_/*N*_B_)	27/143	12/59	88/142	15/83	18/121	28/141
Black women (%)	18.9%	20.3%	29.8% ^A^	18.1%	14.9%	19.7%
Test statistic value	1.92 ^χ2^	1.96 ^χ2^	1.41 ^z^	0.54 *^z^*	1.94 *^t^*	1.68 *^z^*
*p* value *W* vs. *B*	0.056	0.161	0.159	0.587	0.054	0.092
Difference ∆_B-W_	10.8%	11.9%	17.1%	4.5%	15.0%	9.9%
Total	*N*_All_ = 234	*N*_1:1_ = 118	*N*_All_ = 230	*N*_Un-1:1_ = 114	*N*_psmatch2_ = 230	*N*_xtmelogit_ = 229

Notes: *n*_DW_/*N*_W_ and *n*_DB_/*N*_B_ are the numbers of depressed (D) and total Black (B) and White (W) women; tests done: ^χ2^ = chi-squared; *t* = *t*-test; *z* = *z*-test; ^A^: numbers are estimates for a married woman of average age who is unemployed and has less than high school education.

## Supplementary Materials

The following are available online at http://www.mdpi.com/2076-3425/8/12/207/s1.

## References

[1] National Academies of Sciences, Engineering, and Medicine (2018). Building Sustainable Financing Structures for Population Health. Insights from Non-Health Sectors: Proceedings of a Workshop.

[2] US Department of Health and Human Services, Office of Disease Prevention and Health Promotion Healthy People 2020. https://www.healthypeople.gov.

[3] Solar O., Irwin A. A conceptual framework for action on the social determinants of health. http://www.who.int/sdhconference/resources/ConceptualframeworkforactiononSDH_eng.pdf.

[4] Marmot M., Commission on Social Determinants of Health (2007). Achieving health equity: From root causes to fair outcomes. Lancet.

[5] Naimi A.I., Kaufman J.S. (2015). Counterfactual theory in social epidemiology: Reconciling analysis and action for the social determinants of health. Curr. Epidemiol. Rep..

[6] Kaufman J.S., Cooper R.S., McGee D.L. (1997). Socioeconomic status and health in blacks and whites: The problem of residual confounding and the resiliency of race. Epidemiology.

[7] Assari S., Moazen-Zadeh E. (2016). Ethnic variation in the cross-sectional association between domains of depressive symptoms and clinical depression. Fron. Psychiatry.

[8] Coman E.N., Iordache E., Schensul J.J., Coiculescu I. (2013). Comparisons of CES-D depression scoring methods in two older adults ethnic groups. The emergence of an ethnic-specific brief three-item CES-D scale. Int. J. Geriatr. Psychiatry.

[9] Regier D.A., Narrow W.E., Rae D.S. (1990). The epidemiology of anxiety disorders: The epidemiologic catchment area (ECA) experience. J. Psychiatr. Res..

[10] Goldberg D. (2016). On the Very Idea of Health Equity. J. Public Health Manag. Pract..

[11] McLaughlin K.A., Lane R.D., Bush N.R. (2016). Introduction to the Special Issue of Psychosomatic Medicine: Mechanisms Linking Early-Life Adversity to Physical Health. Psychosom. Med..

[12] Font S.A., Maguire-Jack K. (2016). Pathways from childhood abuse and other adversities to adult health risks: The role of adult socioeconomic conditions. Child Abuse Negl..

[13] Assari S. (2014). Additive effects of anxiety and depression on body mass index among blacks: Role of ethnicity and gender. Int. Cardiovasc. Res. J..

[14] Macintyre S., Ellaway A., Cummins S. (2002). Place effects on health: How can we conceptualise, operationalise and measure them?. Soc. Sci. Med..

[15] Lantos P.M., Hoffman K., Permar S.R., Jackson P., Hughes B.L., Kind A., Swamy G. (2017). Neighborhood Disadvantage is Associated with High Cytomegalovirus Seroprevalence in Pregnancy. J. Racial Ethn. Health Dispar..

[16] Bernard P., Charafeddine R., Frohlich K.L., Daniel M., Kestens Y., Potvin L. (2007). Health inequalities and place: A theoretical conception of neighbourhood. Soc. Sci. Med..

[17] Zapata Moya A.R., Navarro Yáñez C.J. (2017). Impact of area regeneration policies: Performing integral interventions, changing opportunity structures and reducing health inequalities. J. Epidemiol. Community Health.

[18] Stewart R., Lindesay J., Abou-Saleh M.T., Katona C., Kumar A. (2011). The epidemiology of depression and anxiety. Principles and Practice of Geriatric Psychiatry.

[19] Bucci M., Marques S.S., Oh D., Harris N.B. (2016). Toxic Stress in Children and Adolescents. Adv. Pediatr..

[20] Jencks C., Mayer S.E., Lynn L.E., McGeary M.G.H. (1990). The social consequences of growing up in a poor neighborhood. Inner-City Poverty in the United States.

[21] Harding D.J. (2003). Counterfactual models of neighborhood effects: The effect of neighborhood poverty on dropping out and teenage pregnancy. Am. J. Sociol..

[22] Crowder K., South S.J. (2011). Spatial and temporal dimensions of neighborhood effects on high school graduation. Soc. Sci. Res..

[23] Garbarski D. (2015). Racial/ethnic disparities in midlife depressive symptoms: The role of cumulative disadvantage across the life course. Adv. Life Course Res..

[24] Levine M.E., Crimmins E.M. (2014). Evidence of accelerated aging among African Americans and its implications for mortality. Soc. Sci. Med..

[25] Neyman J. (1990). On the application of probability theory to agricultural experiments. Essay on principles. Section 9. Stat. Sci..

[26] Highered I. The Numbers and the Arguments on Asian Admissions. https://www.insidehighered.com/admissions/article/2017/08/07/look-data-and-arguments-about-asian-americans-and-admissions-elite.

[27] VanderWeele T.J., Hernán M.A., Berzuini C., Dawid P., Bernardinelli L. (2012). Causal effects and natural laws: Towards a conceptualization of causal counterfactuals for non-manipulable exposures with application to the effects of race and sex. Causality: Statistical Perspectives and Applications.

[28] Pearl J., Mackenzie D. (2018). The Book of Why: The New Science of Cause and Effect.

[29] Bell C.N., Thorpe R.J., Bowie J.V., LaVeist T.A. (2018). Race disparities in cardiovascular disease risk factors within socioeconomic status (SES) strata. Ann. Epidemiol..

[30] Coman E.N., Weeks M.R., Yanovitzky I., Iordache E., Barbour R., Coman M.A., Huedo-Medina T.B. (2012). The Impact of Information About the Female Condom on Female Condom Use Among Males and Females from a US Urban Community. AIDS Behav..

[31] Yanovitzky I., Zanutto E., Hornik R. (2005). Estimating causal effects of public health education campaigns using propensity score methodology. Eval. Progr. Plan..

[32] Cochran W.G. (1950). The comparison of percentages in matched samples. Biometrika.

[33] Morello-Frosch R., Shenassa E.D. (2006). The Environmental “Riskscape” and Social Inequality: Implications for Explaining Maternal and Child Health Disparities. Environ. Health Perspect..

[34] Adler N.E., Rehkopf D.H. (2008). U.S. Disparities in Health: Descriptions, Causes, and Mechanisms. Annu. Rev. Public Health.

[35] National Academies of Sciences, Engineering, and Medicine (2017). The Root Causes of Health Inequity (Ch. 3). Communities in Action: Pathways to Health Equity.

[36] Robert Wood Johnson Foundation Commission to Build a Healthier America Beyond Health Care: New Directions for a Healthier America. https://www.rwjf.org/en/library/research/2009/04/beyond-health-care.html.

[37] Wu Z.H., Tennen H., Hosain G.M.M., Coman E., Cullum J., Berenson A.B. (2016). Stress Mediates the Relationship Between Past Drug Addiction and Current Risky Sexual Behaviour Among Low-income Women. Stress Health.

[38] Coman E.N., Wu H. (2018). Examining Differential Resilience Mechanisms by Comparing ‘Tipping Points’ of the Effects of Neighborhood Conditions on Anxiety by Race/Ethnicity. Healthcare.

[39] Coman E. Pregnancy and Mental Health among Black and White Women, V1 ed.. https://dataverse.harvard.edu/dataset.xhtml?persistentId=doi:10.7910/DVN/9XPEPJ.

[40] Kessler R.C., Andrews G., Mroczek D., Ustun B., Wittchen H.U. (1998). The World Health Organization composite international diagnostic interview short-form (CIDI-SF). Int. J. Methods Psychiatr. Res..

[41] Association A.P., Association A.P. (2000). Diagnostic and Statistical Manual of Mental Disorders.

[42] Carver C.S., White T.L. (1994). Behavioral inhibition, behavioral activation, and affective responses to impending reward and punishment: The BIS/BAS Scales. J. Person. Soc. Psychol..

[43] Cagney K.A., Glass T.A., Skarupski K.A., Barnes L.L., Schwartz B.S., de Leon C.F.M. (2009). Neighborhood-level cohesion and disorder: Measurement and validation in two older adult urban populations. J. Gerontol. Ser. B: Psychol. Sci. Soc. Sci..

[44] Yanovitzky I., Hornik R., Zanutto E., Hayes A., Slater M., Snyder L. (2008). Estimating causal effects in observational studies: The propensity score approach. The Sage Sourcebook of Advanced Data Analysis Methods for Communication Research.

[45] Cochran W.G. (1953). Matching in analytical studies. Am. J. Public Health Nations Health.

[46] Agresti A. (2008). An Introduction to Categorical Data Analysis.

[47] Pearl J. (2009). Causality: Models, Reasoning, and Inference.

[48] Kaufman J.S., Kaufman S. (2001). Assessment of Structured Socioeconomic Effects on Health. Epidemiology.

[49] Stata Corp (2017). Stata Statistical Software: Release 15.

[50] Coman E.N., Picho K., McArdle J.J., Villagra V., Dierker L., Iordache E. (2013). The paired t-test as a simple latent change score model. Front. Quant. Psychol. Meas..

[51] McArdle J.J., Collins L., Horn J.L. (1991). Comments on “latent variable models for studying difference and changes”. Best Methods for the Analysis of Change.

[52] Keyes C.L. (2009). The Black–White paradox in health: Flourishing in the face of social inequality and discrimination. J. Person..

[53] Barnes D.M., Keyes K.M., Bates L.M. (2013). Racial differences in depression in the United States: How do subgroup analyses inform a paradox?. Soc. Psychiatry Psychiatr. Epidemiol..

[54] Nichols A. (2007). Causal inference with observational data. Stata J..

[55] Muthén L.K., Muthén B.O. (1998–2017). Mplus User’s Guide.

[56] Arbuckle J. (2014). AMOS 23 User’s Guide.

[57] Kaufman J.S. (2008). Dissecting disparities. Med. Decis. Mak..

[58] Mahoney J. (2008). Toward a unified theory of causality. Comp. Political Stud..

[59] Marshall A. (1890). Principles of Political Economy.

[60] Heckman J., Pinto R. (2014). Causal Analysis after Haavelmo. Econom. Theory.

[61] Hoff P.D., Raftery A.E., Handcock M.S. (2002). Latent space approaches to social network analysis. J. Am. Stat. Assoc..

[62] Pearl J. (2015). Causes of Effects and Effects of Causes. Sociol. Methods Res..

[63] Pearl J. (2015). Trygve Haavelmo and the emergence of causal calculus. Econom. Theory.

[64] Zhang J., Bareinboim E. Fairness in Decision-Making–The Causal Explanation Formula. Proceedings of the 32nd AAAI Conference on Artificial Intelligence.

[65] Pearl J. (2009). Letter to the editor: Remarks on the method of propensity score. Stat. Med..

[66] Do M.P., Kincaid D.L. (2006). Impact of an Entertainment-Education Television Drama on Health Knowledge and Behavior in Bangladesh: An Application of Propensity Score Matching. J. Health Commun..

[67] Ray K.N., Chari A.V., Engberg J., Bertolet M., Mehrotra A. (2015). Disparities in time spent seeking medical care in the United States. JAMA Internal Med..

[68] Elwert F., Morgan S.L. (2013). Graphical Causal Models. Handbook of Causal Analysis for Social Research.

[69] Kessler R.C., Greenberg D.F. (1981). Linear Panel Analysis: Models of Quantitative Change.

[70] De Haan A., Prinzie P., Sentse M., Jongerling J. (2018). Latent difference score modeling: A flexible approach for studying informant discrepancies. Psychol. Assess..

[71] Kievit R.A., Brandmaier A.M., Ziegler G., van Harmelen A.-L., de Mooij S.M.M., Moutoussis M., Goodyer I.M., Bullmore E., Jones P.B., Fonagy P. (2018). Developmental cognitive neuroscience using latent change score models: A tutorial and applications. Dev. Cogn. Neurosci..

[72] Assari S. (2018). Health disparities due to diminished return among black Americans: Public policy solutions. Soc. Issues Policy Rev..

[73] Mouzon D.M. (2014). Relationships of choice: Can friendships or fictive kinships explain the race paradox in mental health?. Soc. Sci. Res..

