# Supplementary material for: Exploring Causes of Depression and Anxiety Health Disparities (HD) by Examining Differences between 1:1 Matched Individuals
Source: Brain Sci. 2018 Nov 28;8(12):207. doi: 10.3390/brainsci8120207 (PMC6315655; doi:10.3390/brainsci8120207)
Supplement: Supplementary file 1 [file brainsci-08-00207-s001.pdf]

RAW differences in depression = dsm_mder; blvswh== 0 = White, blvswh== 1=Black)

```
RECODE of |       dsm_mder
   blvswh |         0          1 |     Total
-----------+----------------------+----------
         0 |       116         27 |       143
           |     81.12      18.88 |    100.00
-----------+----------------------+----------
         1 |        64         27 |        91
           |     70.33      29.67 |    100.00
-----------+----------------------+----------
     Total |       180         54 |       234
           |     76.92      23.08 |    100.00

          Pearson chi2(1) =   3.6468   Pr = 0.056
```

T-test for depression by White/Black
. ttest dsm_mder , by(blvswh)

```
Two-sample t test with equal variances
------------------------------------------------------------------------------
   Group |     Obs        Mean    Std. Err.   Std. Dev.   [95% Conf. Interval]
---------+--------------------------------------------------------------------
       0 |      91    .2967033    .0481514    .4593354    .2010421    .3923645
       1 |     143    .1888112    .0328421    .3927342    .1238886    .2537338
---------+--------------------------------------------------------------------
combined |     234    .2307692    .0276019    .4222282     .176388    .2851505
---------+--------------------------------------------------------------------
    diff |            .1078921    .0562976               -.0030277    .2188119
------------------------------------------------------------------------------
    diff = mean(0) - mean(1)                                      t =   1.9165
Ho: diff = 0                                     degrees of freedom =      232

    Ha: diff < 0                 Ha: diff != 0                 Ha: diff > 0
 Pr(T < t) = 0.9717         Pr(|T| > |t|) = 0.0565          Pr(T > t) = 0.0283
```

McNemar test for the paired Black/White N=61 pairs for depression

```
-----------
      Total |         61      100.00
. mcci 3 16 9 31

                 | Controls               |
Cases            |   Exposed   Unexposed  |      Total
-----------------+------------------------+------------
         Exposed |         3          16  |         19
       Unexposed |         9          31  |         40
-----------------+------------------------+------------
           Total |        12          47  |         59

McNemar's chi2(1) =      1.96    Prob > chi2 = 0.1615
Exact McNemar significance probability       = 0.2295

Proportion with factor
        Cases       .3220339
        Controls    .2033898     [95% Conf. Interval]
                   ---------     --------------------
        difference  .1186441     -.0616215   .2989096
        ratio       1.583333      .8273921   3.029935
        rel. diff.  .1489362     -.0434179   .3412902

        odds ratio  1.777778      .7397548   4.564309   (exact)
```

2ND OPTION:
* McNemar can be run in MATCHED WIDE=Horizontal data: * Paired and Matched Binary Data
http://health.bsd.uchicago.edu/thisted/hs327/Handouts/0717-Matching.pdf

```
. mcc dsm_mder0 dsm_mder1

                 | Controls               |
Cases            |   Exposed   Unexposed  |      Total
-----------------+------------------------+------------
         Exposed |         3          16  |         19
       Unexposed |         9          31  |         40
-----------------+------------------------+------------
           Total |        12          47  |         59

McNemar's chi2(1) =      1.96    Prob > chi2 = 0.1615
Exact McNemar significance probability       = 0.2295

Proportion with factor
        Cases       .3220339
        Controls    .2033898     [95% Conf. Interval]
                   ---------     --------------------
        difference  .1186441     -.0616215   .2989096
        ratio       1.583333      .8273921   3.029935
        rel. diff.  .1489362     -.0434179   .3412902

        odds ratio  1.777778      .7397548   4.564309   (exact)
```

symmetry dsm_mder0 dsm_mder1

```
0         |     1 dsm_mder
dsm_mder  |   0      1    Total
----------+--------------------
        0 |  31      9     40
        1 |  16      3     19
          |
    Total |  47     12     59
-------------------------------
                                             chi2     df      Prob>chi2
------------------------------------------------------------------------
Symmetry (asymptotic)                |       1.96      1         0.1615
Marginal homogeneity (Stuart-Maxwell) |      1.96      1         0.1615
------------------------------------------------------------------------
```

Logit regression with covariates (DV is dsm_mder, blvswh== 0 = White, blvswh== 1=Black)
. logit dsm_mder ageC i.employ4 i.educ_cat i.mar_cat if blvswh== 0, or

```
Iteration 4:   log likelihood = -49.478486
Logistic regression                               Number of obs     =         88
                                                  LR chi2(9)        =       7.87
                                                  Prob > chi2       =     0.5474
Log likelihood = -49.478486                       Pseudo R2         =     0.0737

-----------------------------------------------------------------------------------------------
                     dsm_mder | Odds Ratio   Std. Err.      z    P>|z|     [95% Conf. Interval]
------------------------------+----------------------------------------------------------------
                         ageC |   .9575861   .0837906    -0.50   0.620     .8066704    1.136736
                              |
                      employ4 |
                    homemaker |   .7343438   .5571957    -0.41   0.684     .1659724    3.249099
                    part-time |   .1581924   .1338212    -2.18   0.029     .0301385    .8303296
                     fulltime |   .3891729   .2426198    -1.51   0.130     .1146788    1.320694
                              |
                     educ_cat |
                 Grade = 12   |    1.51423   .9128101     0.69   0.491     .4645898    4.935308
               f Grade > 12   |   1.060714   .8848526     0.07   0.944     .2067863    5.440949
                              |
                      mar_cat |
                       co-hab |   1.349861    .967305     0.42   0.675     .3313764    5.498657
    not married w/ boyfriend  |    1.27237   1.078574     0.28   0.776     .2415792    6.701431
no not married w/o boyfriend  |   2.253617   1.804069     1.02   0.310     .4693265    10.82144
                              |
```

```
                     _cons |    .424818   .3112403    -1.17   0.243     .1010591    1.785791
------------------------------------------------------------------------------------------
```

Logit regression with covariates (DV is dsm_mder, blvswh== 0 = White, blvswh== 1=Black)
. logit dsm_mder ageC i.employ4 i.educ_cat i.mar_cat if blvswh== 1, or

```
Iteration 0:   log likelihood = -69.072652

Iteration 4:   log likelihood = -60.692839

Logistic regression                             Number of obs     =        142
                                                LR chi2(9)        =      16.76
                                                Prob > chi2       =     0.0526
Log likelihood = -60.692839                     Pseudo R2         =     0.1213

------------------------------------------------------------------------------------------
                  dsm_mder | Odds Ratio   Std. Err.      z    P>|z|     [95% Conf. Interval]
---------------------------+--------------------------------------------------------------
                      ageC |   1.159488    .082431     2.08   0.037     1.008677    1.332847
                           |
                   employ4 |
                 homemaker |   .9943897   .8468647    -0.01   0.995     .1873428    5.278082
                 part-time |   .7829729   .5568095    -0.34   0.731     .1942748    3.155563
                  fulltime |   .5859883   .3243795    -0.97   0.334      .198015    1.734123
                           |
                  educ_cat |
                Grade = 12 |   .5488244    .306535    -1.07   0.283     .1836587    1.640043
              f Grade > 12 |   2.084921   1.283636     1.19   0.233     .6237713    6.968735
                           |
                   mar_cat |
                    co-hab |   .2968817   .2766206    -1.30   0.192      .047805    1.843714
    not married w/ boyfriend |   .2370076   .1958496    -1.74   0.081     .0469221    1.197145
no not married w/o boyfriend |   .2996834   .2654097    -1.36   0.174     .0528214    1.700261
                           |
                     _cons |   .8851557   .7644324    -0.14   0.888      .162896     4.80982
------------------------------------------------------------------------------------------
```

Logit regression with covariates (DV is dsm_mder, blvswh== 0 = White, blvswh== 1=Black)
. logit dsm_mder whvsbl ageC i.employ4 i.educ_cat i.mar_cat , or

```
Iteration 0:   log likelihood = -124.15427
Iteration 1:   log likelihood = -117.59841
Iteration 2:   log likelihood = -117.43767
Iteration 3:   log likelihood =  -117.4375
Iteration 4:   log likelihood =  -117.4375

Logistic regression                             Number of obs     =        230
                                                LR chi2(10)       =      13.43
                                                Prob > chi2       =     0.2004
Log likelihood =  -117.4375                     Pseudo R2         =     0.0541

------------------------------------------------------------------------------------------
                  dsm_mder | Odds Ratio   Std. Err.      z    P>|z|     [95% Conf. Interval]
---------------------------+--------------------------------------------------------------
                    whvsbl |    1.64538   .5818554     1.41   0.159     .8227271     3.29061
                      ageC |   1.061899   .0523098     1.22   0.223     .9641672    1.169537
                           |
                   employ4 |
                 homemaker |   .9870626   .5317445    -0.02   0.981     .3433922    2.837259
                 part-time |   .4524338   .2322648    -1.54   0.122     .1654154    1.237469
                  fulltime |   .5622227   .2212096    -1.46   0.143      .260015    1.215677
                           |
                  educ_cat |
                Grade = 12 |   .9037925   .3438931    -0.27   0.790     .4287326    1.905246
              f Grade > 12 |   1.380755   .6539803     0.68   0.496     .5457002     3.49365
                           |
                   mar_cat |
                    co-hab |   .9197583   .5068748    -0.15   0.879     .3123059    2.708739
    not married w/ boyfriend |   .6335744   .3507736    -0.82   0.410     .2140604    1.875248
no not married w/o boyfriend |    .985657   .5496839    -0.03   0.979     .3303889    2.940534
                           |
```

```
                 _cons |   .3708185    .2111687    -1.74   0.081     .1214593    1.132119
------------------------------------------------------------------------------
```

```
. logit dsm_mder, or  /* check THE 'BASELINE GROUP ODDS */

Iteration 0:   log likelihood = -126.40777
Iteration 1:   log likelihood = -126.40777

Logistic regression                             Number of obs     =      234
LR chi2(0)        = -0.00
Prob > chi2       = .
Log likelihood = -126.40777                     Pseudo R2         =      -0.0000


dsm_mder        Odds   Std. Err.      z     P>z     [95% Conf.     Interval]

_cons          .3    .0465475    -7.76   0.000     .2213351  .4066232
```

```
. logit dsm_mder if whvsbl==0, or  /* whvsbl==0=White whvsbl==1=Black  */

Iteration 0:   log likelihood = -69.282725
Iteration 1:   log likelihood = -69.282725

Logistic regression                             Number of obs     =        143
                                                LR chi2(0)        =       0.00
                                                Prob > chi2       =          .
Log likelihood = -69.282725                     Pseudo R2         =     0.0000

------------------------------------------------------------------------------
    dsm_mder |       Odds   Std. Err.      z    P>|z|     [95% Conf. Interval]
-------------+----------------------------------------------------------------
       _cons |   .2327586   .0497351    -6.82   0.000     .1531172    .3538243
------------------------------------------------------------------------------
```

```
. logit dsm_mder whvsbl, or   /* TO SEE THE ORs or how much more ODDS Blacks than ODDS Whites  */

Iteration 0:   log likelihood = -126.40777
Iteration 1:   log likelihood = -124.62703
Iteration 2:   log likelihood = -124.61483
Iteration 3:   log likelihood = -124.61483

Logistic regression                             Number of obs     =        234
                                                LR chi2(1)        =       3.59
                                                Prob > chi2       =     0.0583
Log likelihood = -124.61483                     Pseudo R2         =     0.0142

------------------------------------------------------------------------------
    dsm_mder | Odds Ratio   Std. Err.      z    P>|z|     [95% Conf. Interval]
-------------+----------------------------------------------------------------
      whvsbl |     1.8125   .5683272     1.90   0.058      .980337    3.351048
       _cons |   .2327586   .0497351    -6.82   0.000     .1531172    .3538243
------------------------------------------------------------------------------
```

```
. logit dsm_mder if whvsbl==1, or  /* whvsbl==0=White whvsbl==1=Black  */

Iteration 0:   log likelihood = -55.332102
Iteration 1:   log likelihood = -55.332102

Logistic regression                             Number of obs     =         91
                                                LR chi2(0)        =      -0.00
                                                Prob > chi2       =          .
Log likelihood = -55.332102                     Pseudo R2         =    -0.0000

------------------------------------------------------------------------------
    dsm_mder |       Odds   Std. Err.      z    P>|z|     [95% Conf. Interval]
-------------+----------------------------------------------------------------
       _cons |    .421875   .0968128    -3.76   0.000     .2690595     .661484
```

```
------------------------------------------------------------------------------
```

```
. logit dsm_mder whvsbl if dyadMMM==., or  /* TO SEE THE 'BASELINE=CONTROL GROUP ODDS */
```

```
Iteration 0:   log likelihood =  -55.91914
Iteration 1:   log likelihood =  -55.77586
Iteration 2:   log likelihood = -55.775434
Iteration 3:   log likelihood = -55.775434

Logistic regression                               Number of obs     =        114
                                                  LR chi2(1)        =       0.29
                                                  Prob > chi2       =     0.5919
Log likelihood = -55.775434                       Pseudo R2         =     0.0026

------------------------------------------------------------------------------
    dsm_mder | Odds Ratio   Std. Err.      z    P>|z|     [95% Conf. Interval]
-------------+----------------------------------------------------------------
      whvsbl |   1.322222   .6818057     0.54   0.588     .4812616    3.632685
       _cons |   .2205882   .0629247    -5.30   0.000     .1261158    .3858291
------------------------------------------------------------------------------
```

```
. ttest dsm_mder  if  match61==1, by(blvswh)
```

```
Two-sample t test with equal variances
------------------------------------------------------------------------------
   Group |     Obs        Mean    Std. Err.   Std. Dev.   [95% Conf. Interval]
---------+--------------------------------------------------------------------
       0 |      60    .3333333    .0613716    .4753827     .210529    .4561377
       1 |      60          .2    .0520756    .4033756     .095797     .304203
---------+--------------------------------------------------------------------
combined |     120    .2666667    .0405379    .4440708    .1863975    .3469358
---------+--------------------------------------------------------------------
    diff |            .1333333    .0804882               -.0260551    .2927218
------------------------------------------------------------------------------
    diff = mean(0) - mean(1)                                      t =   1.6566
Ho: diff = 0                                     degrees of freedom =      118

    Ha: diff < 0                 Ha: diff != 0                 Ha: diff > 0
 Pr(T < t) = 0.9499         Pr(|T| > |t|) = 0.1003          Pr(T > t) = 0.0501
```

```
. ttest anx0  if  match61==0, by(blvswh)
```

```
Two-sample t test with equal variances
------------------------------------------------------------------------------
   Group |     Obs        Mean    Std. Err.   Std. Dev.   [95% Conf. Interval]
---------+--------------------------------------------------------------------
       0 |      23    15.13043    .4231649    2.029428    14.25284    16.00803
       1 |      60       13.75     .261055    2.022123    13.22763    14.27237
---------+--------------------------------------------------------------------
combined |      83    14.13253    .2311168    2.105574    13.67277    14.59229
---------+--------------------------------------------------------------------
    diff |            1.380435    .4964021                .3927503    2.368119
------------------------------------------------------------------------------
    diff = mean(0) - mean(1)                                      t =   2.7809
Ho: diff = 0                                     degrees of freedom =       81

    Ha: diff < 0                 Ha: diff != 0                 Ha: diff > 0
 Pr(T < t) = 0.9966         Pr(|T| > |t|) = 0.0067          Pr(T > t) = 0.0034
```

```
. ttest disomean , by(blvswh)
```

```
Two-sample t test with equal variances
------------------------------------------------------------------------------
   Group |     Obs        Mean    Std. Err.   Std. Dev.   [95% Conf. Interval]
---------+--------------------------------------------------------------------
       0 |      56   -.1525456    .0768839    .5753464   -.3066244    .0015332
       1 |      85     .183892    .0750029    .6914928    .0347405    .3330435
```

```
---------+--------------------------------------------------------------------
combined |     141    .0502714    .0561303    .6665106   -.0607013    .1612441
---------+--------------------------------------------------------------------
    diff |           -.3364376    .1115323               -.5569568   -.1159184
------------------------------------------------------------------------------
    diff = mean(0) - mean(1)                                      t =  -3.0165
Ho: diff = 0                                     degrees of freedom =      139

    Ha: diff < 0                 Ha: diff != 0                 Ha: diff > 0
 Pr(T < t) = 0.0015         Pr(|T| > |t|) = 0.0030          Pr(T > t) = 0.9985
```

IN MATCHED DATA n=61 DYADIC

```
. ttest disomean0==disomean1

Paired t test
------------------------------------------------------------------------------
Variable |     Obs        Mean    Std. Err.   Std. Dev.   [95% Conf. Interval]
---------+--------------------------------------------------------------------
disome~0 |      21   -.2024293    .1319519    .6046794    -.477676    .0728175
disome~1 |      21    .1933944    .1353469    .6202374   -.0889343    .4757231
---------+--------------------------------------------------------------------
    diff |      21   -.3958237    .1993738    .9136453   -.8117101    .0200627
------------------------------------------------------------------------------
     mean(diff) = mean(disomean0 - disomean1)                     t =  -1.9853
 Ho: mean(diff) = 0                              degrees of freedom =       20

 Ha: mean(diff) < 0           Ha: mean(diff) != 0           Ha: mean(diff) > 0
 Pr(T < t) = 0.0305         Pr(|T| > |t|) = 0.0610          Pr(T > t) = 0.9695
```

```
dsm_mder0==in WHITE women
dsm_mder0==in BLACK women
ttest dsm_mder0==dsm_mder1
Paired t test
------------------------------------------------------------------------------
Variable |     Obs        Mean    Std. Err.   Std. Dev.   [95% Conf. Interval]
---------+--------------------------------------------------------------------
dsm_md~0 |      59    .3220339    .0613537    .4712667    .1992211    .4448467
dsm_md~1 |      59    .2033898    .0528535    .4059752    .0975921    .3091876
---------+--------------------------------------------------------------------
    diff |      59    .1186441    .0840415    .6455349   -.0495833    .2868714
------------------------------------------------------------------------------
     mean(diff) = mean(dsm_mder0 - dsm_mder1)                     t =   1.4117
 Ho: mean(diff) = 0                              degrees of freedom =       58

 Ha: mean(diff) < 0           Ha: mean(diff) != 0           Ha: mean(diff) > 0
 Pr(T < t) = 0.9183         Pr(|T| > |t|) = 0.1634          Pr(T > t) = 0.0817
```

```
. ttest anx00c==anx01c

Paired t test
------------------------------------------------------------------------------
Variable |     Obs        Mean    Std. Err.   Std. Dev.   [95% Conf. Interval]
---------+--------------------------------------------------------------------
  anx00c |      24     .092949    .4332741    2.122601   -.8033468    .9892448
  anx01c |      24    .3910253    .3180367    1.558055   -.2668837    1.048934
---------+--------------------------------------------------------------------
    diff |      24   -.2980763    .5229486    2.561914   -1.379878    .7837253
------------------------------------------------------------------------------
     mean(diff) = mean(anx00c - anx01c)                           t =  -0.5700
 Ho: mean(diff) = 0                              degrees of freedom =       23

 Ha: mean(diff) < 0           Ha: mean(diff) != 0           Ha: mean(diff) > 0
 Pr(T < t) = 0.2871         Pr(|T| > |t|) = 0.5742          Pr(T > t) = 0.7129
```

```
. ttest disomean0=disomean1
```

```
Paired t test
------------------------------------------------------------------------------
Variable |     Obs        Mean    Std. Err.   Std. Dev.   [95% Conf. Interval]
---------+--------------------------------------------------------------------
disome~0 |      21   -.2024293    .1319519    .6046794    -.477676    .0728175
disome~1 |      21    .1933944    .1353469    .6202374   -.0889343    .4757231
---------+--------------------------------------------------------------------
    diff |      21   -.3958237    .1993738    .9136453   -.8117101    .0200627
------------------------------------------------------------------------------
     mean(diff) = mean(disomean0 - disomean1)                     t =  -1.9853
 Ho: mean(diff) = 0                              degrees of freedom =       20

 Ha: mean(diff) < 0           Ha: mean(diff) != 0           Ha: mean(diff) > 0
 Pr(T < t) = 0.0305         Pr(|T| > |t|) = 0.0610          Pr(T > t) = 0.9695
```

The 61 matching results. With 4 covariates, thinner bars are White wome, gray bars are Black women.

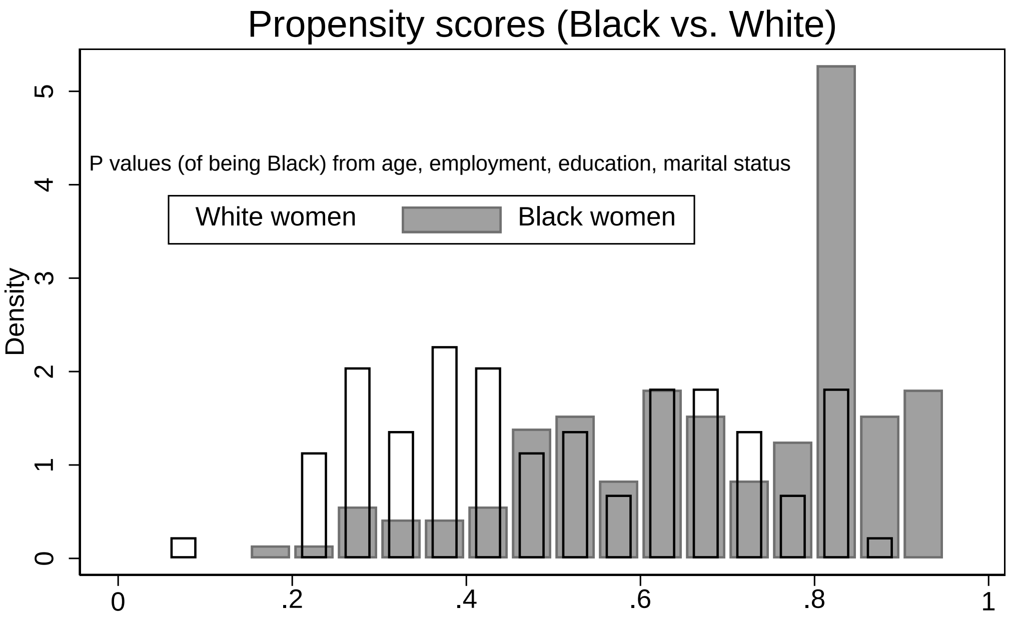


```
. Classic regression:
reg BIS_Anxiety_AVG0 blvswh age dr_tp_30d0 i.employ4 i.educ_cat i.mar_cat /*income*/
clust15_on4 has 15 clusters, 1 with no cases of white women:
      educ |        blvswh
   marital |         0          1 |     Total
-----------+----------------------+----------
         1 |         6          2 |         8
         3 |         9          4 |        13
         4 |         6          3 |         9
         5 |        10          3 |        13
         6 |         9          4 |        13
         7 |         5         10 |        15
         8 |         6         11 |        17
         9 |         3          6 |         9
        10 |         8         13 |        21
        11 |         8         11 |        19
        12 |         6          6 |        12
        13 |         3          9 |        12
```

```
        14 |         8         38 |        46 
        15 |         1         11 |        12 
        16 |         0         12 |        12 
-----------+----------------------+----------
     Total |        88        143 |       231 
```

“teffects uses gmm to compute the final results. Like any optimization routine, it takes starting values and then iterates to the solution. If you look at the iteration log you will find something like:” Stata discussion

Clustered regression
. xtmelogit dsm_mder if whvsbl== 0|| clust15_on4: , or //

```
Mixed-effects logistic regression               Number of obs      =       229
Group variable: clust15_on4                     Number of groups   =        15

                                                Obs per group:
                                                              min =          8
                                                              avg =       15.3
                                                              max =         45

Integration points =   7                        Wald chi2(1)       =      2.83
Log likelihood =   -122.227                     Prob > chi2        =    0.0923

------------------------------------------------------------------------------
    dsm_mder | Odds Ratio   Std. Err.      z    P>|z|     [95% Conf. Interval]
-------------+----------------------------------------------------------------
      blvswh |   .5765182   .1886098    -1.68   0.092     .3036265    1.094678
       _cons |   .4158645   .1013208    -3.60   0.000      .257968    .6704058
------------------------------------------------------------------------------
Note: _cons estimates baseline odds (conditional on zero random effects).

------------------------------------------------------------------------------
  Random-effects Parameters  |   Estimate   Std. Err.     [95% Conf. Interval]
-----------------------------+------------------------------------------------
clust15_on4: Identity        |
                   sd(_cons) |   .2012963   .3619906      .0059309    6.832023
------------------------------------------------------------------------------
LR test vs. logistic model: chibar2(01) = 0.09        Prob >= chibar2 = 0.3799
----
```

Clustered regression 141 Blacks only:

```
Mixed-effects logistic regression               Number of obs      =       141
Group variable: clust15_on4                     Number of groups   =        15

                                                Obs per group:
                                                              min =          2
                                                              avg =        9.4
                                                              max =         37

Integration points =   7                        Wald chi2(0)       =          .
Log likelihood = -67.948908                     Prob > chi2        =          .

------------------------------------------------------------------------------
    dsm_mder |       Odds   Std. Err.      z    P>|z|     [95% Conf. Interval]
-------------+----------------------------------------------------------------
       _cons |   .2447559   .0688085    -5.01   0.000     .1410698    .4246511
------------------------------------------------------------------------------
------------------------------------------------------------------------------
  Random-effects Parameters  |   Estimate   Std. Err.     [95% Conf. Interval]
-----------------------------+------------------------------------------------
clust15_on4: Identity        |
                   sd(_cons) |   .5866403   .3329702      .1928589    1.784448
------------------------------------------------------------------------------
LR test vs. logistic model: chibar2(01) = 1.82        Prob >= chibar2 = 0.0884
```

Clustered regression
88 Whites only:

```
Mixed-effects logistic regression               Number of obs      =        88
```

```
Group variable: clust15_on4                     Number of groups  =         14

                                                Obs per group:
                                                              min =          1
                                                              avg =        6.3
                                                              max =         10

Integration points =   7                        Wald chi2(0)      =          .
Log likelihood = -53.412798                     Prob > chi2       =          .

------------------------------------------------------------------------------
    dsm_mder |       Odds   Std. Err.      z    P>|z|     [95% Conf. Interval]
-------------+----------------------------------------------------------------
       _cons |   .4193548   .0979807    -3.72   0.000     .2652782    .6629209
------------------------------------------------------------------------------

------------------------------------------------------------------------------
  Random-effects Parameters  |   Estimate   Std. Err.     [95% Conf. Interval]
-----------------------------+------------------------------------------------
clust15_on4: Identity        |
                   sd(_cons) |   1.90e-09    .524181             0           .
------------------------------------------------------------------------------
LR test vs. logistic model: chibar2(01) = 0.00        Prob >= chibar2 = 1.0000
```

```
Clustered regression
xtmelogit dsm_mder whvsbl|| dyadMMM: , or /* */ //
```

```
Refining starting values:

Iteration 0:   log likelihood = -69.711133
Iteration 1:   log likelihood = -68.564934
Iteration 2:   log likelihood = -68.244595
Performing gradient-based optimization:
Iteration 0:   log likelihood = -68.244595
  Iteration 3:   log likelihood = -68.214996

Mixed-effects logistic regression               Number of obs     =        120
Group variable: dyadMMM                         Number of groups  =         61

                                                Obs per group:
                                                              min =          1
                                                              avg =        2.0
                                                              max =          2

Integration points =   7                        Wald chi2(1)      =       2.68
Log likelihood = -68.214996                     Prob > chi2       =     0.1015
------------------------------------------------------------------------------
    dsm_mder | Odds Ratio   Std. Err.      z    P>|z|     [95% Conf. Interval]
-------------+----------------------------------------------------------------
      whvsbl |          2   .8465616     1.64   0.102     .8724342    4.584873
       _cons |        .25   .0806872    -4.30   0.000     .1328055    .4706132
------------------------------------------------------------------------------
Note: _cons estimates baseline odds (conditional on zero random effects).

------------------------------------------------------------------------------
  Random-effects Parameters  |   Estimate   Std. Err.     [95% Conf. Interval]
-----------------------------+------------------------------------------------
dyadMMM: Identity            |
                   sd(_cons) |   1.18e-08   .7595531             0           .
------------------------------------------------------------------------------
LR test vs. logistic model: chibar2(01) = 0.00        Prob >= chibar2 = 1.0000
```

```
. logit dsm_mder if whvsbl== 0 , or /* Black *NO clustering WITHIN W or B!!!*/
```

```
Iteration 0:   log likelihood = -69.282725
Iteration 1:   log likelihood = -69.282725
Logistic regression                             Number of obs     =        143
                                                LR chi2(0)        =       0.00
                                                Prob > chi2       =          .
Log likelihood = -69.282725                     Pseudo R2         =     0.0000
```

```
------------------------------------------------------------------------------
    dsm_mder |       Odds   Std. Err.      z    P>|z|     [95% Conf. Interval]
-------------+----------------------------------------------------------------
       _cons |   .2327586   .0497351    -6.82   0.000     .1531172    .3538243
------------------------------------------------------------------------------
```

```
. logit dsm_mder if whvsbl== 1 , or /* White *NO clustering WITHIN W or B!!!*/
```

```
Iteration 0:   log likelihood = -55.332102
Iteration 1:   log likelihood = -55.332102

Logistic regression                               Number of obs     =         91
                                                  LR chi2(0)        =      -0.00
                                                  Prob > chi2       =          .
Log likelihood = -55.332102                       Pseudo R2         =    -0.0000

------------------------------------------------------------------------------
    dsm_mder |       Odds   Std. Err.      z    P>|z|     [95% Conf. Interval]
-------------+----------------------------------------------------------------
       _cons |    .421875   .0968128    -3.76   0.000     .2690595     .661484
------------------------------------------------------------------------------
```

```
Model for the unmatchable into dyads:
```

```
. tab whvsbl if dyadMMM== .

  RECODE of |
     blvswh |      Freq.     Percent        Cum.
------------+-----------------------------------
          0 |         84       73.04       73.04
          1 |         31       26.96      100.00
------------+-----------------------------------
      Total |        115      100.00
```

```
. logit dsm_mder whvsbl if dyadMMM== ., or
```

```
Iteration 0:   log likelihood =  -55.91914
Iteration 1:   log likelihood =  -55.77586
Iteration 2:   log likelihood = -55.775434
Iteration 3:   log likelihood = -55.775434

Logistic regression                               Number of obs     =        114
                                                  LR chi2(1)        =       0.29
                                                  Prob > chi2       =     0.5919
Log likelihood = -55.775434                       Pseudo R2         =     0.0026

------------------------------------------------------------------------------
    dsm_mder | Odds Ratio   Std. Err.      z    P>|z|     [95% Conf. Interval]
-------------+----------------------------------------------------------------
      whvsbl |   1.322222   .6818057     0.54   0.588     .4812616    3.632685
       _cons |   .2205882   .0629247    -5.30   0.000     .1261158    .3858291
------------------------------------------------------------------------------
```

```
. logit dsm_mder if dyadMMM== . & whvsbl==0, or /* Black */
```

```
Iteration 0:   log likelihood = -39.216493
Iteration 1:   log likelihood = -39.216493

Logistic regression                               Number of obs     =         83
                                                  LR chi2(0)        =       0.00
                                                  Prob > chi2       =          .
Log likelihood = -39.216493                       Pseudo R2         =     0.0000

------------------------------------------------------------------------------
    dsm_mder |       Odds   Std. Err.      z    P>|z|     [95% Conf. Interval]
-------------+----------------------------------------------------------------
       _cons |   .2205882   .0629247    -5.30   0.000     .1261158    .3858291
```

```
. logit dsm_mder  if dyadMMM== . & whvsbl==1, or /*White*/
```

```
Iteration 0:   log likelihood =  -16.55894
```

```
Iteration 1:   log likelihood =  -16.55894

Logistic regression                             Number of obs     =         31
                                                LR chi2(0)        =      -0.00
                                                Prob > chi2       =          .
Log likelihood =  -16.55894                     Pseudo R2         =    -0.0000

------------------------------------------------------------------------------
    dsm_mder |       Odds   Std. Err.      z    P>|z|     [95% Conf. Interval]
-------------+----------------------------------------------------------------
       _cons |   .2916667    .125289    -2.87   0.004     .1256732      .67691
------------------------------------------------------------------------------
```

```
. logit dsm_mder whvsbl ageC i.employ4 i.educ_cat i.mar_cat if dyadMMM== ., or
```

```
Iteration 0:   log likelihood = -55.703674
Iteration 1:   log likelihood = -52.174923
Iteration 2:   log likelihood =  -52.00878
Iteration 3:   log likelihood = -52.008154
Iteration 4:   log likelihood = -52.008154

Logistic regression                             Number of obs     =        113
                                                LR chi2(10)       =       7.39
                                                Prob > chi2       =     0.6881
Log likelihood = -52.008154                     Pseudo R2         =     0.0663

----------------------------------------------------------------------------------------------
                    dsm_mder | Odds Ratio   Std. Err.      z    P>|z|     [95% Conf. Interval]
-----------------------------+----------------------------------------------------------------
                      whvsbl |     .96099   .7075814    -0.05   0.957     .2269735    4.068764
                        ageC |   1.116355    .084001     1.46   0.144     .9632815    1.293754
                             |
                     employ4 |
                   homemaker |     .25405   .3082017    -1.13   0.259     .0235657    2.738782
                   part-time |   .7328493   .5064547    -0.45   0.653      .189134    2.839617
                    fulltime |   .5107403    .304783    -1.13   0.260     .1585797    1.644949
                             |
                    educ_cat |
                  Grade = 12 |   .8048461    .459135    -0.38   0.704     .2631079    2.462022
                f Grade > 12 |   .8972994   .8325809    -0.12   0.907     .1455893    5.530256
                             |
                     mar_cat |
                      co-hab |   .6675242   .6286212    -0.43   0.668     .1054079    4.227279
    not married w/ boyfriend |   .3097302   .3120383    -1.16   0.245     .0429971    2.231147
no not married w/o boyfriend |   .5255433   .5676935    -0.60   0.551     .0632613    4.365952
                             |
                       _cons |   .8295378   .8699049    -0.18   0.859     .1062217     6.47827
----------------------------------------------------------------------------------------------
```

```
Propensity matching
psmatch2 whvsbl age i.employ4 i.educ_cat i.mar_cat ,  outcome (dsm_mder) common ate  /*-2.48/0.41
306 of 313 matched!*/
```

```
Probit regression                               Number of obs     =        230
                                                LR chi2(9)        =      44.66
                                                Prob > chi2       =     0.0000
Log likelihood = -130.69464                     Pseudo R2         =     0.1459

----------------------------------------------------------------------------------------------
                      whvsbl |      Coef.   Std. Err.      z    P>|z|     [95% Conf. Interval]
-----------------------------+----------------------------------------------------------------
                         age |  -.0684868   .0288463    -2.37   0.018    -.1250245    -.011949
                             |
                     employ4 |
                   homemaker |   .3183986   .3183482     1.00   0.317    -.3055524    .9423496
                   part-time |   .4044835   .2604296     1.55   0.120    -.1059492    .9149162
                    fulltime |   .0249496   .2162795     0.12   0.908    -.3989504    .4488496
                             |
                    educ_cat |
                  Grade = 12 |  -.0459396   .2115003    -0.22   0.828    -.4604725    .3685934
```

```
             f Grade > 12 |   .6464417    .270198     2.39   0.017     .1168635     1.17602
                          |
                  mar_cat |
                   co-hab |  -.3515176   .3170399    -1.11   0.268    -.9729043    .2698692
  not married w/ boyfriend |  -1.469985   .3125524    -4.70   0.000    -2.082577   -.8573934
no not married w/o boyfriend |  -.9199494    .317925    -2.89   0.004    -1.543071   -.2968279
                          |
                    _cons |   1.890958   .7454051     2.54   0.011     .4299907    3.351925
------------------------------------------------------------------------------------------------
There are observations with identical propensity score values.
The sort order of the data could affect your results.
Make sure that the sort order is random before calling psmatch2.
----------------------------------------------------------------------------------------
        Variable     Sample |    Treated     Controls   Difference         S.E.   T-stat
----------------------------+-----------------------------------------------------------
        dsm_mder  Unmatched | .295454545   .190140845    .1053137   .056956018     1.85
                        ATT | .298850575   .149425287   .149425287    .07715606     1.94
                        ATU | .181818182   .314049587   .132231405            .        .
                        ATE |                           .139423077            .        .
----------------------------+-----------------------------------------------------------
Note: S.E. does not take into account that the propensity score is estimated.

 psmatch2: |   psmatch2: Common
 Treatment |        support
assignment | Off suppo  On suppor |     Total
-----------+----------------------+----------
 Untreated |        21        121 |       142
   Treated |         1         87 |        88
-----------+----------------------+----------
     Total |        22        208 |       230
```

```
. teffects psmatch (dsm_mder) (whvsbl age i.employ4 i.educ_cat i.mar_cat ) LOGIT default
```

```
Treatment-effects estimation                   Number of obs      =        230
Estimator      : propensity-score matching     Matches: requested =          1
Outcome model  : matching                                    min =          1
Treatment model: logit                                       max =          5
------------------------------------------------------------------------------
             |              AI Robust
    dsm_mder |      Coef.   Std. Err.      z    P>|z|     [95% Conf. Interval]
-------------+----------------------------------------------------------------
ATE          |
       whvsbl |
   (1 vs 0)  |   .1507246   .1047891     1.44   0.150    -.0546583    .3561075
```

Illustration of the LDS/LCS model in AMOS with 2 'difference/disparities' variables: Neighborhood Disorder=Neigh. And Anxiety =Anx (for B=Black and W=White women)Amos

Neighborhood disorder: 0=for White women; **1=for Black women**
Anxiety: 0=for White women; 1=for Black women

Input: 1's and 0's are FIXED parameters
LDS_61_NeighDisorder_toAnxiety_v5.amw

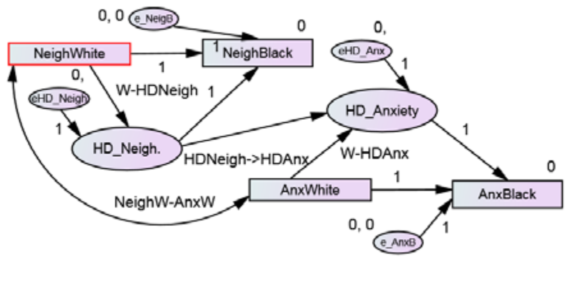

Output graphical results of estimation
LDS_61_NeighDisorder_toAnxiety_1.AmosOutput
LDS_61_NeighDisorder_toAnxiety_1_AmosOutput.pdf

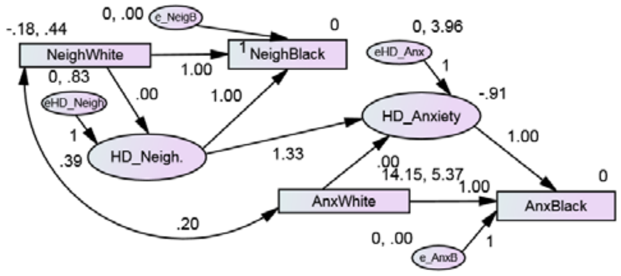

Regression Weights: (Matched61pairs - AllEst)

	StandEst.	UnstandEst.	S.E.	C.R.	P	Label
X1Neigh → X2Anx	.519	1.331	.498	2.674	.007	HDNeigh->HDAnx

Mplus output of HD(Neighborhood disorder)->HD(Anxiety)
lds_anxhd on neighhd_propgr0.out

```
define:

  x10=disomean0 ; ! Whites Neighborhood disorder
  x11=disomean1 ; ! Blacks Neighborhood disorder

  x20=anx00c ; ! Whites Anxiety
  x21=anx01c ; ! Blacks Anxiety

  model:
  ! X1 part
  x1LdsBW by x11;
      x11@0;
      [x11@0];
      x11 on x10 @1;
      [x1LdsBW];
        x1LdsBW ;
  x10 with x1LdsBW@0; !this can be relaxed: estimated

  ! X2 part
  x2LdsBW by x21;
      x21@0;
      [x21@0];
      x21 on x20 @1;
      [x2LdsBW];
        x2LdsBW ;
  x20 with x2LdsBW@0;   !this can be relaxed: estimated

  ! ~~~ ******************Bivariate **************
  x2LdsBW on x1LdsBW;
   X20      WITH    X1LDSBW @0; !Mplus estimates them otherwise
  output: sampstat stand      tech4 tech9;

R-SQUARE
     Latent                                           Two-Tailed
    Variable        Estimate       S.E.  Est./S.E.    P-Value

    X2LDSBW            0.412      0.175      2.353      0.019
```

MODEL RESULTS

```
                                                      Two-Tailed
                    Estimate       S.E.  Est./S.E.    P-Value

 X1LDSBW  BY
    X11                1.000      0.000    999.000    999.000
 X2LDSBW  BY
    X21                1.000      0.000    999.000    999.000
 X2LDSBW  ON
    X1LDSBW            1.813      0.439      4.129      0.000
 X11      ON
    X10                1.000      0.000    999.000    999.000
 X21      ON
    X20                1.000      0.000    999.000    999.000

 X10      WITH
    X1LDSBW            0.000      0.000    999.000    999.000
 X20      WITH
    X2LDSBW            0.000      0.000    999.000    999.000
```

```
    X1LDSBW            0.000      0.000    999.000    999.000
 X20      WITH
    X10                0.304      0.254      1.197      0.231
 Means
    X10               -0.015      0.100     -0.146      0.884
    X20               -0.095      0.346     -0.275      0.783
    X1LDSBW            0.313      0.149      2.099      0.036
 Intercepts
    X11                0.000      0.000    999.000    999.000
    X21                0.000      0.000    999.000    999.000
    X2LDSBW           -0.349      0.398     -0.876      0.381
 Variances
    X10                0.406      0.090      4.521      0.000
    X20                5.410      1.127      4.802      0.000
    X1LDSBW            0.678      0.177      3.832      0.000
 Residual Variances
    X11                0.000      0.000    999.000    999.000
    X21                0.000      0.000    999.000    999.000
    X2LDSBW            3.183      1.085      2.935      0.003
STANDARDIZED MODEL RESULTS
STDYX Standardization
                                                    Two-Tailed
                    Estimate       S.E.  Est./S.E.    P-Value
 X1LDSBW  BY
    X11                0.791      0.050     15.776      0.000
 X2LDSBW  BY
    X21                0.707      0.057     12.305      0.000
 X2LDSBW  ON
    X1LDSBW            0.642      0.136      4.706      0.000
 X11      ON
    X10                0.612      0.065      9.452      0.000
 X21      ON
    X20                0.707      0.057     12.299      0.000
 X10      WITH
    X1LDSBW            0.000      0.000    999.000    999.000
 X20      WITH
    X2LDSBW            0.000      0.000    999.000    999.000
    X1LDSBW            0.000      0.000    999.000    999.000
 X20      WITH
    X10                0.205      0.161      1.271      0.204
 Means
    X10               -0.023      0.158     -0.146      0.884
    X20               -0.041      0.148     -0.275      0.783
    X1LDSBW            0.380      0.192      1.976      0.048
 Intercepts
    X11                0.000      0.000    999.000    999.000
    X21                0.000      0.000    999.000    999.000
    X2LDSBW           -0.150      0.168     -0.893      0.372
 Variances
    X10                1.000      0.000    999.000    999.000
    X20                1.000      0.000    999.000    999.000
    X1LDSBW            1.000      0.000    999.000    999.000
 Residual Variances
    X11                0.000    999.000    999.000    999.000
    X21                0.000    999.000    999.000    999.000
    X2LDSBW            0.588      0.175      3.360      0.001
```

Mplus output of HD(Neighborhood disorder)->HD(Depression)
lds_deprhd on neighhd_propgr0.out

```
define:
  y0=dsm_mder0 ; ! Whites Depression
  y1=dsm_mder1 ; ! Blacks Depression
```

```
  x10=disomean0 ; ! Whites Neighborhood disorder
  x11=disomean1 ; ! Blacks Neighborhood disorder

  model:
  ! X1 part
  x1LdsBW by x11;
      x11@0;
      [x11@0];
      x11 on x10 @1;
      [x1LdsBW];
        x1LdsBW ;
  x10 with x1LdsBW @0;

  ! Y part
  yLdsBW by y1;
      y1@0;
      [y1@0];
      y1 on y0 @1;
      [yLdsBW];
        yLdsBW ;
  y0 with yLdsBW @0;

  ! ~~~ ******************Bivariate **************

  yLdsBW on x1LdsBW;
   Y0       WITH    X1LDSBW @0; !Mplus estimates them otherwise
```

R-SQUARE

Latent Variable	Estimate	S.E.	Est./S.E.	Two-Tailed P-Value
YLDSBW	0.038	0.076	0.505	0.614

MODEL RESULTS

	Estimate	S.E.	Est./S.E.	Two-Tailed P-Value
X1LDSBW BY				
X11	1.000	0.000	999.000	999.000
YLDSBW BY				
Y1	1.000	0.000	999.000	999.000
YLDSBW ON				
X1LDSBW	0.154	0.153	1.006	0.314
X11 ON				
X10	1.000	0.000	999.000	999.000
Y1 ON				
Y0	1.000	0.000	999.000	999.000
X10 WITH				
X1LDSBW	0.000	0.000	999.000	999.000
Y0 WITH				
YLDSBW	0.000	0.000	999.000	999.000
X1LDSBW	0.000	0.000	999.000	999.000
X10 WITH				
Y0	-0.008	0.046	-0.164	0.870
Means				
Y0	0.332	0.061	5.493	0.000
X10	-0.157	0.101	-1.551	0.121
X1LDSBW	0.385	0.153	2.524	0.012
Intercepts				
Y1	0.000	0.000	999.000	999.000
X11	0.000	0.000	999.000	999.000
YLDSBW	-0.183	0.104	-1.751	0.080

```
 Variances
    Y0                 0.221      0.040      5.503      0.000
    X10                0.402      0.088      4.545      0.000
    X1LDSBW            0.659      0.172      3.827      0.000
 Residual Variances
    Y1                 0.000      0.000    999.000    999.000
    X11                0.000      0.000    999.000    999.000
    YLDSBW             0.390      0.075      5.198      0.000
STANDARDIZED MODEL RESULTS
STDYX Standardization
                                                    Two-Tailed
                    Estimate       S.E.  Est./S.E.    P-Value

 X1LDSBW  BY
    X11                0.788      0.051     15.528      0.000
 YLDSBW   BY
    Y1                 0.805      0.037     22.038      0.000
 YLDSBW   ON
    X1LDSBW            0.196      0.194      1.010      0.313
 X11      ON
    X10                0.616      0.065      9.475      0.000
 Y1       ON
    Y0                 0.594      0.049     12.002      0.000
 X10      WITH
    X1LDSBW            0.000      0.000    999.000    999.000
 Y0       WITH
    YLDSBW             0.000      0.000    999.000    999.000
    X1LDSBW            0.000      0.000    999.000    999.000
 X10      WITH
    Y0                -0.025      0.155     -0.164      0.870
 Means
    Y0                 0.707      0.144      4.912      0.000
    X10               -0.247      0.161     -1.531      0.126
    X1LDSBW            0.475      0.197      2.406      0.016
 Intercepts
    Y1                 0.000      0.000    999.000    999.000
    X11                0.000      0.000    999.000    999.000
    YLDSBW            -0.287      0.163     -1.759      0.079
 Variances
    Y0                 1.000      0.000    999.000    999.000
    X10                1.000      0.000    999.000    999.000
    X1LDSBW            1.000      0.000    999.000    999.000
 Residual Variances
    Y1                 0.000    999.000    999.000    999.000
    X11                0.000    999.000    999.000    999.000
    YLDSBW             0.962      0.076     12.658      0.000
```
